# Subtelomeric Rearrangements in Patients with Recurrent Miscarriage 

**DOI:** 10.22074/ijfs.2018.5260

**Published:** 2018-06-20

**Authors:** Amani Hajlaoui, Wafa Slimani, Molka Kammoun, Amira Sallem, Fathi El Amri, Anouar Chaieb, Mohamed Bibi, Ali Saad, Soumaya Mougou-Zerelli

**Affiliations:** 1 Department of Cytogenetic and Reproductive Biology, Farhat Hached University Hospital, Sousse, Tunisia; 2 Private pediatrician, Tunis, Tunisia; 3 Department of Obstetrics and Gynecology, Farhat Hached University Hospital, Sousse, Tunisia

**Keywords:** Chromosomal Aberration, Fluorescent In Situ Hybridization, Intellectual Disability, Translocation, Spon- taneous Abortion

## Abstract

**Background:**

The subtelomeric rearrangements are increasingly being investigated in cases of idiopathic intellectual disabilities (ID) and congenital abnormalities (CA) but are also thought to be responsible for unexplained recurrent miscarriage (RM). Such rearrangements can go unnoticed through conventional cytogenetic techniques and are undetectable even with high-resolution molecular cytogenetic techniques such as array comparative genomic hybridization (aCGH), especially when DNA of the stillbirth or families are not available. The aim of the study is to evaluate the rate of subtelomeric rearrangements in patients with RM.

**Materials and Methods:**

In this cross-sectional study, fluorescent in situ hybridization (FISH), based on ToTelVysion telomeric probes, was undertaken for 21 clinically normal couples exhibiting a “normal” karyotype with at least two abortions. Approximately 62% had RM with a history of stillbirth or CA/ID while the other 38% had only RM.

**Results:**

FISH detected one cryptic rearrangement between chromosomes 3q and 4p in the female partner of a couple (III:4) [46,XX,ish t(3;4)(q28-,p16+;p16-,q28+)(D3S4559+,D3S4560-,D4S3359+; D3S4560+, D4S3359- ,D4S2930+)] who presented a history of RM and family history of ID and CA. Analysis of the other family members of the woman showed that her sisters (III:6 and III:11) and brother (III:8) were also carriers of the same subtelomeric translocation t(3;4)(q28;p16).

**Conclusion:**

We conclude that subtelomeric FISH should be undertaken in couples with RM especially those who not only have abortions but also have had at least one child with ID and/or CA, or other clinically recognizable syndromes. For balanced and cryptic anomalies, subtelomeric FISH still remains the most suitable and effective tool in characterising such chromosomal rearrangements in RM couples.

## Introduction

IRecurrent miscarriage (RM), one of the most frus. trating problems faced by both patients and clinicians, is recently defined by the American Society for Reproductive Medicine as the miscarriage of two or moreconsecutive pregnancies in the first or early secondtrimester of gestation (1).

When conventional cytogenetic techniques are used, balanced chromosomal anomalies are detected inabout 5% of RM cases (2). Such rearrangements mayresult in meiotic errors and chromosomal nondisjunc. tion, leading to the production of unbalanced gametes. The resulting unbalanced chromosomal constitution ingametes may lead to the birth of malformed children, RM or infertility (3). Several studies have shown that the presence of stillborn and/or live born malformedchildren, in addition to spontaneous abortion, increases the probability for a couple that one partner is a carrier of a balanced translocation (4, 5). Cryptic subtelomeric translocations, which would be missed byconventional techniques (6), may also be frequent insuch cases.

In the past decade, subtelomeric rearrangements have been shown to be implicated in congenital malformations and intellectual disabilities (ID) (7, 8). Therefore, high resolution cytogenetic techniquessuch as subtelomeric fluorescent in situ hybridization(FISH) screening and array comparative genomic hy. bridization (aCGH) were developed and became reference tools for rearrangement screening in ID and congenital abnormalities (CA) cases (9). This need for technological advancement was due to cryptic anomalies being missed by conventional cytogenetic techniques because of their small size and similar banding patterns. Furthermore, because of their quantitative pattern, even revolutionary tools such as aCGH and multiplex ligation-dependent probe multiplex amplification (MLPA) cannot detect balanced rearrangements. Interestingly, only a few studies have proven the usefulness of subtelomeric FISH screening in these cases (7, 10, 11). 

Nevertheless, the effectiveness of such a tool in RM cases is still unclear and the exact incidence of such rearrangements remains uncertain. In this study, we screened subtelomeric regions in 21 couples having experienced two or more spontaneous abortions with or without stillbirth and/or children with CA to examine the rate of cryptic subtelomeric translocations in RM.

## Materials and Methods

This cross-sectional study was undertaken in the Department of Cytogenetic and Reproductive Biology at Farhat Hached University Teaching Hospital (Sousse, Tunisia). We selected 21 clinically normal couples, from 01/07/2012 to 31/07/2013, based on the inclusion criteria of having at least two abortions and exhibiting “normal” karyotypes. These couples had normal endocrine function and had no medically assisted procreation attempt in the study period. The women had normal ovarian function, normal genital organs and had no anterior toxic exposure, trauma, radiotherapy, chemotherapy, chronic diseases or medications. The local Ethics Board approved the present study (IRB00008931) and all patients gave informed consent for this study.

### Fluorescent in situ hybridization

R banding karyotyping on peripheral blood lymphocyte cultures was carried out systematically. FISH based on Vysis ToTelVysion Multi-Color FISH Probe Kit (Abbott ® Molecular Inc., Des Plaines, USA) was performed for the 21 couples according to the standard protocol. This kit contained 41 TelVysion probes which were specific to subtelomeric regions of chromosomes 1-12 and 16-20, subtelomeres of the q arm of acrocentric chromosomes and pseudo-autosomal region subtelomeres (Xp/Yp and Xq/Yq). For each chromosome, we analyzed at least ten cells and in case of translocations, more metaphases were considered.

## Results

**Fig.1:**
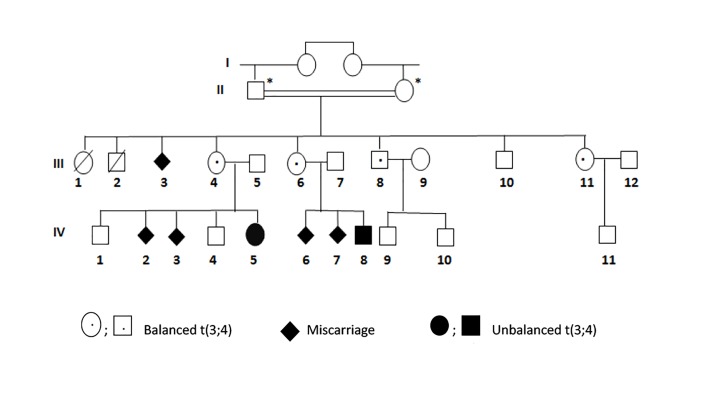
Family pedigree. The black arrow points to the couple number 21 (III: 4 and III: 5) in this study. *; Not available for analysis.

Among the 21 selected couples, only one cryptic subtelomeric translocation was found in the female partner of the 21st couple who were referred to the Obstetrics and Gynecology Department and had a history of two abortions. The pedigree of this couple is illustrated in Figure 1. Around 62% had RM with history of stillbirth or CA/ ID and the other 38% had only RM. Using i. TelVysion 3p Spectrum Green (D3S4559) and TelVysion 3q Spectrum Orange (D3S4560) for chromosome 3, and ii. TelVysion 4p Spectrum Green (D4S3359) and TelVysion 4q Spectrum Orange (D4S2930) for chromosome 4, subtelomeric FISH analysis of this patient (III:4) showed a subtelomeric translocation between the long and short arms of chromosomes 3 and 4 respectively. Her chromosomal formula was 46,XX,ish t (3;4) (q28-p16+;p16,q28+) (D3S4559+, D3S4560-D4S3359+; D3S4560+, D4S3359, D4S2930+).

**Fig.2:**
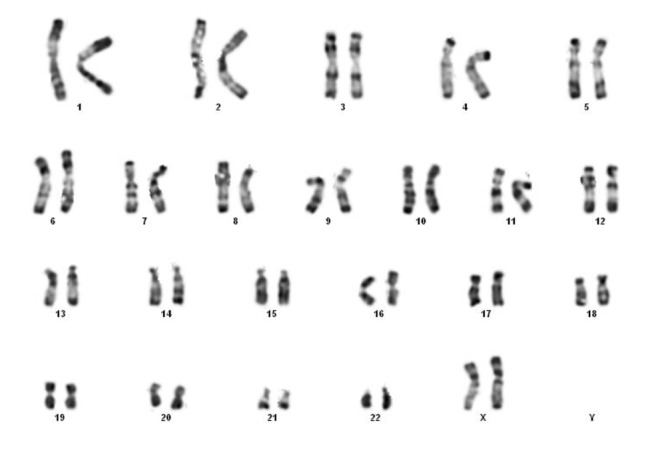
Karyotype of the mother (III: 4).

**Fig.3:**
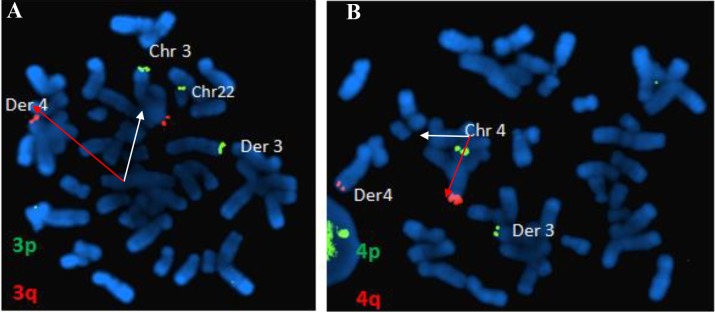
FISH on metaphase spread prepared from the women’s (III:4) blood with Mix 3 and Mix 4 from Vysis ToTelVysion Kit. A. Mixture 3: chromosome 3 (p spectrum green and q spectrum orange) and chromosome 22 (q spectrum green + spectrum orange), LCI bcr (22q11) spectrum aqua. The red signal of the 3q telomere probe is observed on the normal 3q chromosome (white arrow) and on the short arm of the derivative chromosome 4 (red arrow) and B. Mixture 4: chromosome 4 (p spectrum green and q spectrum orange) and chromosome 21 (q spectrum green+spectrum orange), LCI bcr (21q22) spectrum aqua. Similarly, as expected, the green signal of the probe specific for telomere 4p is observed on normal chromosome 4 (white arrow) and derivative 3 (red arrow).

The partial karyotype of this patient (III:4) showed apparently normal banding pattern of chromosomes 3 and 4 (Fig .2). FISH results are shown in Figure 3. Investigation of other family members of III:4 showed that her sisters (III:6 and III:11) and brother (III:8) were also carriers of the same translocation t(3;4)(q28;p16).

With this molecular information ignored by the family, two years later, patient III:4 requested consultation after giving birth to a daughter (IV:5) with congenital malformations and ID. The classical cytogenetic analysis of IV:5 showed that she inherited the same translocation in its unbalanced form (46, XX, ish der (4), t(3;4)(q28;p16) from her mother (results not shown).

A review of the literature of screening for subtelomeric rearrangements is summarized in Table 1.

**Table 1:** Review of studies screening subtelomeric regions in patients with recurrent miscarriage


Study	Number of studied cases	Number of subtelomeric rearrangements	Number of miscarriages	Malformed/stillborn child	Translocation	Technique

Wakui et al. (12)	10	5	2 or more	+	46, XY, t (7;16)(q36; q22)	Dual-colour subtelomere FISH
				+	46, XX, t (4; 7)(q35; p15.3)	
				+	46, XX, t(5; 10)(p15.1; p13	
				-	46,XY,t(1;5)(q42;q33)	
				+	46,XY,t(7;13)(q36.2;q34)	
Giardino et al. (13)	1 family with 2 female carriers	1	2	+	46, XX, t(2;16)(q37.3;q24.3)	Multi-subtelomere FISH using the CytocellMultiprobe-T system
Benzacken et al. (14)	114	0	2 or more	NM	-	Multi-subtelomere FISH using the CytocellMultiprobe-T system
Fan and Zhang (15)	80	0	4 or more	NM	-	Multi-subtelomere FISH using the CytocellMultiprobe-T system
Joyce et al. (16)	2	2	2	+	46,XX,t(11;17)(p15.5;p13.3)	Dual-colour subtelomere FISH
			4	+	46,XX, t(11;17)(p15.5;p13.3)	FISH analysis with telomere-specific probes
Yakut et al. (17)	10	2	5 or more	-	46,XY,ish t(3q; 10p)	Subtelomere specific FISH probe
			5 or more	-	46,XX,ish t(20p;?Dp)	Multi-subtelomere FISH using the CytocellMultiprobe-T system
Jalal et al. (18)	53	0	Multiple	NM	-	
Bruyere et al. (19)	1	1	3	+	46,XX, t(2;17) (q37.2; q25)	Multi-subtelomere FISH using the CytocellMultiprobe-T system
Cockwell et al. (20)	100	1	7	-	46,XX t(3:10)(q29;p15.3)	ToTelVysion Multi-Color FISH
Monfort et al. (21)	36	1	7	-	46,XX,t(2;3)	ToTelVysion Multi-Color + Miller-Dieker probe
Primerano et al. (22)	1	1	2	+	46,XX. t(5;17)	ToTelVysion Multi-Color FISH
Present study	42	1	2	+	46, XX, t(3;4)(q28;p16)	
Total	448	15		5-		
				10+		


+; Exist, -; Does not exist, and NM; Not Mentioned.

## Discussion

Humans are characterized by a high rate of embryonic failure at the early stages of development. RM, stillbirths and the birth of children with multiple CA remain the most spectacular varieties of this reproductive failure. The cause of RM remains elusive in approximately 50% of cases, although many studies have attempted to identify the underlying mechanism (23). Interestingly, it has been shown that an unknown proportion of parents who appear chromosomally normal on conventional cytogenetic analysis may carry cryptic subtelomeric rearrangements following malsegregation or recombination at gametogenesis, giving rise to segmental aneuploidy and thus resulting in RM (20, 24). This missingness is because of the same banding pattern at telomeric regions as well as their small size at the 500-550 band level karyotype resolution (19, 25).

 The purpose of the present study was to examine whether RM is associated with subtelomeric rearrangements. Among the 42 individuals tested, one female showed a cryptic translocation between the 3q and 4p arms with distal breakpoints near the telomeres. Consistently, her affected daughter (IV:5) showed inheritance of the same translocation in its unbalanced form (46, XX, der 4,t(3;4) (q28;p16)) based on classical cytogenetic analysis. In this family, it was important to consider that not only a higher risk of RM was observed, but also congenital anomalies were present in subsequent pregnancies of carriers of cryptic rearrangements. Depending on the type of the reciprocal translocation, it has been estimated that the recurrent risk varies from1 to 50% (26, 27). The recurrent risk of the present patient for subsequent pregnancies was estimated to be 25%. Accordingly, genetic counseling should be mandatory after the diagnosis of a cryptic reciprocal translocation. The affected couple should be well informed about subsequent abortions, risks of transmission of the aberration, as well as giving birth to malformed children.

We reviewed the literature and identified eleven studies which had screened for subtelomeric regions in patients with RM. By combining these studies and our present report, 15 out of 448 patients showed subtelomeric translocations based on different sets of telomeric probes. This give a total rate of 3.34% for carriers of cryptic translocations while this rate was 4.76% in this study. Interestingly, these carriers not only had a history of RM, but also had a history of giving birth to children with ID and/or CA, or a clinically recognizable syndrome. This shows the importance of detailing the family history in improving diagnosis and suggesting the appropriate tool of exploration for precision in genetic counseling.

As previously mentioned, both conventional karyotyping and more advanced techniques such as aCGH and MLPA have limitations in detecting subtle genomic aberrations including balanced rearrangements (28, 29). These limitations have been overcome by using subtelomeric FISH. However, the high cost of subtelomeric FISH (due to expensive consumables) makes the clinical application of this technique unjustifiable for all couples presenting with a history of only RM and aCGH seems to be more practical. However, for couples with RM and a family history of stillbirth or children with CA or ID, subtelomeric FISH should be mandatory as it represents the most efficient approach in diagnosing RM couples than any other molecular assay.

## Conclusion

Identification of cryptic subtelomeric translocations is an active area of investigation. This study emphasizes the importance of screening these types of balanced rearrangements with subtelomeric FISH particularly in couples with RM. We conclude that subtelomeric FISH analysis should become mandatory for couples with RM and familial family history of stillbirth or children with CA or ID. This has a great impact when DNA or abortion products are no longer available. In fact, these products are not always accessible due to incompatibility with life of the congenital anomalies besides, in some cases, patient refusal to be tested. In addition, miscarriages and ID remain a somewhat offensive and delicate subject for parents, which makes the recruitment of patients more difficult in familial cases of RM. Furthermore, subtelomeric FISH is required to exclude any cytogenetic cause before searching for other spermatic factors such as sperm aneuploidy, sperm DNA integrity, chromatin packaging and semen parameters as we previously reported. Through this study, we highlight the importance of early clinical identification of such cases toward a more efficient diagnosis of subtelomeric translocations in RM cases.
